# *“My condition is my secret”*: perspectives of HIV positive female sex workers on differentiated service delivery models in Kampala Uganda

**DOI:** 10.1186/s12913-022-07561-x

**Published:** 2022-02-04

**Authors:** Lydia Atuhaire, Constance S. Shumba, Peter S. Nyasulu

**Affiliations:** 1 Division of Epidemiology and Biostatistics, Faculty of Medicine and Health Sciences, Stellenbosch University, Cape Town, South Africa; 2 School of Nursing and Midwifery, Aga Khan University, Nairobi, Kenya; 3 Department of Population Health, Aga Khan University, Nairobi, Kenya; 4 School of Public Health, Faculty of Medicine and Health Sciences, University of the Witwatersrand, Johannesburg, South Africa

**Keywords:** Differentiated care, Female Sex Workers, Kampala, HIV services access

## Abstract

**Background:**

Differentiated service delivery (DSD) models for female sex workers (FSWs) continue to be scaled up with the goal of expanding access to HIV services and treatment continuity. However, little is known about FSWs’ perspectives on their preferences, facilitators, and barriers to the effective utilization of various DSD models.

**Methods:**

We conducted 24 in-depth interviews among FSWs on antiretroviral therapy for at least one year in two drop-in centres and two public health facilities in Kampala, Uganda in January 2021.

**Results:**

The facility-based individual management model was most preferred, due to a wide array of comprehensive health services, privacy, and professional health workers. Community DSD models were physically accessible, but least preferred due to stigmatization and discrimination, lack of privacy and confidentiality, and limited health services offered.

**Conclusion:**

Targeted strategies to reduce stigma and discrimination and the provision of high-quality services have potential to optimise FSWs’ access to HIV services.

## Introduction

The differentiated service delivery (DSD) models have been implemented in many countries for the last six years since the World Health Organisation (WHO) [1] released new antiretroviral therapy (ART) guidelines with DSD models, and a subsequent decision framework on differentiated ART services for Key populations (KPs) [2]. DSD models are a client-centered approach that focuses on the preferences and needs of clients [3]. The approach addresses the contexts and clinical characteristics of clients, and aims to individualize care for client populations using a public health approach [1, 4]. For female sex workers (FSWs), DSD models also aim to address the inequities in access by increasing acceptability, quality, and coverage of HIV services [5, 6]. In Sub-Saharan Africa, DSD models have been rolled out through facility-based and community-based delivery models. These include community drug distribution points (CDDPs) with tailored services provided in hotspots and drop in centres (DICs), and community ART Groups, an example of community client-led ART delivery model (CCLAD) where clients rotate to pick medication from the facility [7, 8].

Uganda’s Ministry of Health [9] adapted the WHO recommendation on DSD models for people living with HIV (PLWHIV), and an implementation guide was developed in 2017 [10]. Currently, five approaches are being implemented including two facility-based models, two community-based models as well as one cross-cutting model. The facility-based models are Facility based individual management (FBIM), and Facility based groups (FBGs), while the community-based models are community client- led ART delivery model (CCLAD) and community drug distribution points (CDDPs). Examples of such drug pick-up points include DICs, mobile outreaches in hot spots, and community pharmacy pick up points [10]. The cross-cutting model is the fast-track drug refill (FTDR). All community-based models, fast track drug refills as well as a few FBG models serve stable clients, while all the unstable clients are provided services through FBIMs. To be categorized as a stable client, one must have spent more than 12 months on ART, demonstrate good adherence of above 95% and is virally suppressed [10].

The benefits of DSD models on service delivery improvement for PLWHIV are not refuted, as indeed, several studies have reported successes especially on increasing HIV diagnosis and attaining desired HIV treatment outcomes [7, 8, 11–13]. While the success of DSD models hinges on continued process evaluations to understand clients’ needs, preferences, and behaviours [2, 14], the reported health outcomes have been mostly linked to the factors related to the health systems organization. The studies that attempted to seek clients’ perspectives on DSD models targeted the general population of PLWHIV [14, 15], and one study mainly focused on clients’ acceptability of Pharmacy‑Based Delivery of Pre‑Exposure Prophylaxis [16]. FSWs have unique challenges with increased risk to HIV acquisition, and therefore assessing their perspectives on how DSD models influence FSWs’ individual and population-level utilization of services is critical. Their insights can potentially contribute to increased access and sustainable comprehensive HIV prevention and treatment services for FSWs. Differentiated service provision, should progressively be informed by FSWs’ individual preferences, as well as contextual factors throughout the service delivery journey [11]. This study explored the perspectives of FSWs on DSD models in order, to guide strategies aimed at increasing access to HIV services and retention in HIV care for FSWs.

## Methods

### Study design

This was a qualitative descriptive study conducted in January 2021 using In-Depth Interviews (IDIs) to explore perspectives of 24 FSWs living with HIV and accessing differentiated service delivery models.

### Study setting

The study was conducted at two public health facilities and two drop-in centres located in Kampala, Uganda. The health facilities provide general health services and run HIV clinics for the general population including FSWs. The drop-in centres provide key population [17] targeted HIV services provided under a PEPFAR grant. The study areas were purposively sampled with the support of Infectious Disease Institute, a PEPFAR implementing partner that governs health facilities and other KP community-based service points in Kampala. The study sample consisted of ART facilities and drop-in centres that have adopted differentiated service delivery models in Kampala and were purposively selected based on the information obtained from the routine DSD data collected through monthly and quarterly National Health Management Information System reports [18]. These facilities were therefore deemed to serve participants with rich information to share about their experiences while receiving services through DSD models for FSWs.

### Participants and the recruitment process

The FSWs were purposively selected if they had been on ART for a minimum of one year and had rich experiences to share about DSD models. For recruitment, we engaged DSD focal persons, FSW peers and DIC managers as gatekeepers to identify potential participants as they accessed ART services. The study team emphasized to the gatekeepers that the interviews about the DSD models did not constitute a staff or facility performance. This prior information was meant to reduce potential for gatekeepers to bias the sample by referring only FSWs that would potentially talk positively about their experiences on DSD models. The gatekeepers introduced the study to FSWs and referred them to the research team who explained the study information in detail. Interviews were then conducted following verbal consent to participate. The research team comprised of the first author who is a PhD Fellow and has an academic background in social sciences with expert knowledge of qualitative research methodology. The first author was assisted by two research assistants with a minimum of five years’ experience in qualitative research methods.

### Data collection and management

We developed an interview guide and collected data on 5 domains: (i) general experiences on HIV services access through DSD models, [19] benefits and challenges of DSD models, (iii) participants’ perceptions of their level of involvement in the choices of DSD models they are assigned to, (iv) the adequacy of DSD model settings, and (v) recommendations for improvement of service delivery in DSD models. The interview guide was framed based on the content in the decision framework on differentiated ART services for KPs [2], and other themes reflected in the relevant published literature on DSD models [6, 20]. Participants’ demographics were captured using close-ended questions. The IDI guide and all consenting documents were translated in Luganda; the local language used in Kampala, back translated into English and two pilot interviews were conducted. The IDIs were conducted in January 2021 in the participants’ preferred language, either English or Luganda and were audio recorded. The interviews took place in private rooms provided by the DICs and facilities, and they took 45–60 min. We followed the principle of data saturation recommended for ensuring rigour in qualitative data analysis [21], implying that we stopped interviews after determining that no new information was emerging. Data saturation was presumed attained when we had interviewed twenty-two [22] participants and determined that no new information was emerging. We then added two [2] more interviews to confirm that we had achieved data saturation before we completely stopped the interviews at twenty-four [24] participants. Data were transcribed verbatim and translated into English. For quality checks, half of the transcripts (twelve) were proofread against the audio, and any missed or misheard words were identified, and corrections were made. Random spot-checks were also performed on all remaining transcripts. The finalized content and the recordings were stored on a password protected and encrypted computer.

### Data analysis

Data analysis was conducted manually using framework analysis [22] and we applied an inductive approach [23] which involved systematically reviewing the code framework, reduction, and interpretation of the data. The coding framework was developed based on the key domains indicated in the interview guide and the aim of the study. After familiarization with the transcripts and field notes, transcripts were coded manually using a code framework developed in excel by the two members of the research team. There were no major conflicts with codes, however, consensus was reached for minor differences. Common themes, patterns and relationships were identified in relation to already coded categories, and this supported data interpretation. Lastly, data were summarized by linking the major themes with the study aim and objective, and possible agreements and contradictions were highlighted.

### Ethics

The study was approved by the Institutional Review Boards of the Uganda Virus Research Institute (UVRI), and Ethics Committee of Faculty of Medicine and Health Sciences of Stellenbosch University, and Uganda National Council of Science and Technology (reference number HS-2665). In addition, a letter of administrative support was obtained from Kampala Capital City Authority health office. All participants were given detailed information about the study and were informed that their participation was voluntary and they could withdraw from the interviews at any time. They also provided verbal informed consent as opposed to written consent, in line with the guidance from the Institutional Review Boards on conducting research among key populations.

## Results

### Sample population

There were 24 participants in the study, of which half 50.0% (12/24) were 20–30 years old. 50% (12/24) of the participants had not completed primary level education, only 16.6% (4/24) had completed secondary education and none of the participants had post-secondary education. Most of the participants 58% (14/24) were either separated or widowed of which the majority 75% (18/24) had between one to four children. Over half of the participants 54% (13/24) had done sex work for one to five years and the majority 66% (16/24) of these were in HIV care for at least one to five years **(**Table 1**).**

**Table 1:** Participant characteristics *N*  = 24

Characteristics	Results	Frequency	Percentage
Average age	31		
Age category	20–30 31–40 41–50	12 11 1	50 45.84 4.16
Education level	None Primary level not completed Primary level completed Secondary level not completed Secondary level completed	6 6 3 5 4	25 25 12.5 20.84 16.66
Marital status	Single Separated Widowed Married	8 12 2 2	33.34 50 8.33 8.33
Number of children	None One Two Three Four	6 4 7 5 2	25 16.66 29.16 20.84 8.33
Period in sex work	1–5 years 6–10 years 11–15 years 16–20 years	13 6 3 2	54.17 25 12.5 8.33
Period in care and ART	1–5 years 6–10 years 11–15 years	16 7 1	66.6 29 4

We describe the themes that emerged from this study and a summary of the codes, categories, and themes **(**Table 2**).** They include: i) DSD models currently accessed by FSW, ii) reasons for FSWs’ preference of specific DSD models, and iii) barriers to FSWs’ uptake of preferred DSD mode.

**Table 2:** Themes, Codes, and Exemplar Quotes of FSW Differentiated Care

Themes	Codes	Exemplar quotes
DSD models currently accessed by FSW	**Facility based DSD model:** •Fast track drug refill •Facility based individual management	**Facility based DSD models** “I come to the facility, and I give in my card, until they call me to see a doctor or nurse. They examine me and If I need some tests, they send me to the laboratory for tests (FBIM)” (26 years FSW, 2 years on ART)
	**Community based DSD models** •Community Client Led ART delivery model •Community Drug Distribution Point, specifically Drop-in Centres	**Community based DSD models** “There is another delivery model which is currently on, where the peers pick drugs for us, or we alternate among us CLLAD members. You just need to give a group member your ART number and they pick drugs for you from the facility” (25 years FSW, 1 year on ART)
DSDs preferred by FSWs and the reasons for preference	**A.Facility based DSD models** **FBIM** •Access to free medication for all illnesses •Privacy and confidentiality **FTDR** •Reduced waiting time **B.Community based model** **Community pharmacy and DICs** •Easy access •Flex and long working hours •Friendly health workers **CCLAD** •Reduced transport costs	**A.Facility based DSD models** “I don’t spend money on any kind of treatment because I get all the health services I may need when I come at the facility. Once I get fever or cough, I still get treatment. I am able to get all the health services I may want as long as I come by myself at the facility” (37 years FSW, 5 years on ART) “I would not want that to happen to me (community members knowing her status). I would rather collect the drugs for myself at the facility, my condition is my secret” (35 years FSW, 3 years on ART) **B. Community based model** “I really feel at peace in this place (DIC), the way the doctors treat us, they handle us with care. They understand the health services that suit us, so we feel free to open up when we have issues” (28 years FSW, 3 years on ART “Another thing is one can easily access the ARTs whenever she feels like since they are in the community. Even if a person forgets to pick her drugs, she can easily go there and pick more ARTs” (32 years FSW, 10 years on ART)
Barriers to FSWs’ uptake of preferred DSD models	**A.Facility based models** •Non-flexible and short working hours •Poor health workers’ attitude •Failure to adapt to the social contexts of FSWs during service delivery	**A.Facility based models** “The challenge I face is that those people (facility staff) stop receiving ART cards at midday. So, when you come past midday, they may not serve you. Because we work at night, we have fatigue in the morning and you may be late” (32 years FSW, 3 years on ART) “They isolate us to the extreme. At my previous facility, there is a doctor who made a statement to me which made me shed tears. Remember I am a strong person now imagine how it would feel for someone who needs a lot of encouragement” (28 years FSW, 3 years on ART) “I faced a challenge one time when the health workers were asking me to bring my partner for testing. I had told them that I am sexually active but had not disclosed that I do sex work, they were stressing me every time I come, may be because I would be having STIs, almost every visit. I felt like shifting from that facility”. (35 years FSW, 3 years on ART)
	**B.Community based models** •Lack of trust and conflicts in groups •Community stigma •Limited-service packages	**B.Community based models** **“**They (CCLAD members) don’t even gossip about our HIV status only, they even talk about your other private things. For example, me, I am married but also do sex work, imagine if someone told my husband and children!!!” (30 years FSW, 4 years on ART) “Something that we do today will affect us in future in that even if you reach a time and quit the sex work job, people will not believe you. So, they may even discriminate against your children and even insult them that they are children of a sex worker. We suffer much criticism from the community members, and this becomes challenging to seek care from the service centre in my community” (28 years FSW, 3 years on ART) “If I attend to these other places where services are just brought to us, I may have fever, or an infection or cough and they fail to give me the treatment. They just prescribe for me the medicine and they tell me to go and buy it, because they don’t carry all medicines, yet I come here knowing that medicine is available” (27 years FSW, 4 years on ART)

Our study indicated that the most accessed differentiated service delivery model types were (i) facility-based individual management [19] fast track drug refills and (iii) community drug distribution points specifically ART access through Drop-in Centres. Participants presented mixed feelings about the DSD models available to them for care access versus the models they would prefer to use. The main reasons for preference of FBIM and FTDR models included access to free comprehensive HIV services, short waiting time and privacy. We found that there were several impediments to accessing some of the DSD models such as community client-led art delivery and outreach-based services. These included lack of confidentiality, lack of trust, and limited-service packages provided in some of these models.

### DSD models currently accessed by FSWs

There were two main types of DSD models accessed by FSWs: facility-based DSD models and community-based DSD models. One type of facility-based DSD model called Facility-Based Individual Management was accessed by FSWs, and another one was a crosscutting DSD model called Fast Track Drug Refill which was also accessed at the facility. On the other hand, the community-based DSD models included Community Client-Led ART Delivery model and Community Drug Distribution Points specifically, Drop-in Centres. Notably, the facility-based DSD models that were not mentioned by participants included Facility-Based Groups. Similarly, Community Pharmacy and other CDDPs such as outreaches and home ART deliveries were not mentioned under the Community Based DSD models. However, some of these were mentioned among the preferred models by the participants.

### Facility-based DSD models accessed by FSWs

In this study, two types of Facility-Based Models dominated the narratives, and these include FBIM and FTDR, a model that either is accessed at the DIC or at facility level. The participants were able to explain the different ways in which they access ART, and we deduced that they were accessing FBIM and FTDR per the following narratives.

Once I reach the facility, I just go straight to the health worker who keeps the ART files. I come with the ART cards for my colleagues and hand them over to that health worker [ Fast track drug refill] (25 years FSW, 4 years on ART).

I come to the facility, and I give in my card, until they call me to see a doctor or nurse. They examine me and If I need some tests, they send me to the laboratory for tests (FBIM) (26 years FSW, 2 years on ART).

### Community based DSD models accessed by FSWs

Like the facility-based models, participants mentioned the community DSD models they were enrolled in by providing accounts of how they receive care. Based on their explanations we were able to determine that the community based DSD models mostly accessed by FSW are CDDPs, specifically DICs and CCLADs as shown in the narratives.

When it is finished (ARVs) I come here (DIC), and I get a refill. I come very early in the morning and I get my drugs and leave. However, I have a doctor here, in case I fail to come to pick my drugs he can pack it properly and put it on the taxi and I pay the conductor when the taxi reaches Masako. (35 years FSW, 10 years on ART).

There is another delivery model which is currently on, where the peers pick drugs for us, or we alternate among us CLLAD members. You just need to give a group member your ART number and they pick drugs for you from the facility (25 years FSW, 1 year on ART).

### Theme 2: DSDs preferred by FSWs and the reasons for preference

#### Access to free comprehensive services from the facility

Participants had strong opinions on accessing HIV care directly from the facility because this provided them with the benefit of accessing free comprehensive health services. This was especially true for those who were receiving care through the FBIM. Further, participants expressed that they benefitted from facility-based models by being attended to by doctors at every visit, being physically examined and accessing laboratory tests, and drugs for other illnesses. Being able to receive a free comprehensive package and see a doctor regularly were highlighted as important aspects of care by the participants as described:

I don’t spend money on any kind of treatment because I get all the health services I may need when I come at the facility. Once I get fever or cough, I still get treatment. I am able to get all the health services I may want as long as I come by myself at the facility (37 years FSW, 5 years on ART).

When I reach the health facility, I get the drugs and if I am suffering any illness, I get to see the doctor and get all the treatment. You see the problem with being at a DIC or picking from a community pharmacy, sometimes these other drugs are not there, and you even don’t get a chance of seeing a doctor (38 years FSW, 2 years on ART).

#### Reduced waiting time at the clinic

The improved efficiency and the ability of participants to get time to do their private work was prominently expressed in this study especially among those that were served through the FTDR at the facility. Participants generally recognised that they now spent less time at the facility, compared to the time before the FTDR had been implemented. This is echoed in the sentiments:

Nowadays I receive quick services which wasn’t the case previously. In the past I would spend a whole day at the facility. Imagine now with COVID lockdown, I would not be working at all, if I wasn’t given my drug quickly, because these days we get customers during the day because of lockdown (27 years FSW, 4 years on ART).

#### Availability of privacy and confidentiality at the facility

Many participants in this study had strong feelings about the privacy the clinic settings provide to them. In addition, they also felt that the facility setting provides an enabling environment for confidentiality and comfort of participants while accessing care without worries of inadvertent disclosure:

I want the one where I am coming to the facility for drugs by myself, because here you are sure no one knows what has brought you there. Even when we are telling our things to nurses no-one is there to listen to your information. See, in the community they see us from wherever they find us, even in places that are not private enough. (32 years FSW, 10 years on ART).

I would not want that to happen to me (community members knowing her status). I would rather collect the drugs for myself at the facility, my condition is my secret (35 years FSW, 3 years on ART).

#### Reasons for preference, specific to community-based DSD models

##### Easy service access

Many participants in this study attributed their preference of community based DSD models to the improved service access they provide. They mentioned that services especially at the DIC are located within their reach, where they work and stay so they did not spend money on transport. They also reported that DICs work on flex time, nurses are available to be contacted on phone and FSWs can come in at any time and access drugs. This is highlighted verbatim as follows:

Another thing is one can easily access the ARTs whenever she feels like since they are in the community. Even if a person forgets to pick her drugs, she can easily go there and pick more ARTs. (32 years FSW, 10 years on ART).

**Participant:** The good thing with this place (DIC), you can easily get support from health workers anytime you may need it which is different from when I go at the facility.

**Interviewer**: What do you mean by getting support anytime?

**Participant:** Here they can even be open up to until late or you can phone call a nurse any time when you have a need (45 years FSW, 7 years on ART).

##### Access to peer support

Participants who showed their preference to receive care in the DICs, had strong opinions on the peer support experienced when accessing ART refills especially relating to the fact that they have a dual burden of living with HIV, as well as engaging in work that makes them face exclusions in the community. In their own words, participants mentioned how meeting healthy-looking colleagues made them feel encouraged and sharing experiences with colleagues made them feel stronger.

Whenever I sit with my fellow female sex workers here (DIC) and they share their HIV experiences, I get encouraged. Sometimes my colleagues share the years they have been on ART, and I also get more determined to take my ART well. Sharing experiences with my colleagues makes me feel relaxed that I am not alone in this situation (35 years FSW, 2 years on ART).

##### Friendly health workers

Many participants mentioned that the friendliness of health workers at DICs reduced the reluctance to self-identify as FSWs to the health workers, and therefore benefitted from appropriate health services:

I really feel at peace in this place (DIC), the way the doctors treat us, they handle us with care. They understand the health services that suit us, so we feel free to open up when we have issues (28 years FSW, 3 years on ART).

If I am unable to pick my drugs on the exact appointment date, I just let the doctors here (DIC) know and they keep my drugs until I pick them without scorning me. Of course, I skip just a few days. This would never happen at these public facilities. Here, I can tell you I am very comfortable (22 years FSW, 2 years on ART).

### Theme 3: Barriers to FSWs’ uptake of preferred DSD models

#### Lack of flexibility in facility based DSD models

In this study many participants expressed difficulties related to access of services through facility-based DSD models, yet they would have preferred to do so. The participants mentioned shortcomings of facility-based models related to failure to adapt to the social contexts in which beneficiaries live. For example, they indicated that facilities have fixed working hours and that they are sometimes pressed to bring their partners for testing which they say may not favour their way of life. In the following statements their concerns are expressed:

The challenge I face is that those people (facility staff) stop receiving ART cards at midday. So, when you come past midday, they may not serve you. Because we work at night, we have fatigue in the morning and you may be late (32 years FSW, 3 years on ART).

I faced a challenge one time when the health workers were asking me to bring my partner for testing. I had told them that I am sexually active but had not disclosed that I do sex work, they were stressing me every time I come, may be because I would be having STIs, almost every visit. I felt like shifting from that facility (35 years FSW, 3 years on ART).

#### Perceived corruption is associated with receipt of quick health services

While access of services through specific DSD models is dependent on whether a client is categorised as stable on ART or not, or the preference of individuals, participants in this study perceived this differently. They indicated that individuals who receive quick services specifically through the FTDR, either paid health workers ‘under the table’ or were favoured because they were personally known to them and were selectively provided with quick health services.

I know those who receive quick health services under FTDR bribe the health workers to get those quicker services. They do it secretly with the health workers. I would rather use the money to buy my children food and follow all the procedures I go through to get my ART than paying money to be enrolled into FTDR (35 years FSW, 3 years on ART).

#### Poor health worker’s attitude

Participants shared some negative experiences encountered with health workers when accessing services at the facility based DSD models. The general view of participants was that HWs had stigmatizing attitudes towards FSWs that needed to be addressed. This is what was echoed in relation to health workers attitude:

If you happen to disclose to them that you do our kind of jobs (sex work), they discuss about you the moment you leave. And the next time you go back they will all want to identify you by what you shared with them while showing a lot of negative attitudes. I think they need to be sensitized to accept our kind of work because here (DIC), I do not face such (27 years FSW, 6 years on ART).

They isolate us to the extreme. At my previous facility, there is a doctor who made a statement to me which made me shed tears. Remember I am a strong person now imagine how it would feel for someone who needs a lot of encouragement (28 years FSW, 3 years on ART).

#### Lack of transport

Lack of transport came out as a significant barrier for routine clinic visits promulgating challenges to access drugs through facility-based models. Participants indicated that this was a constant source of stress and anxiety, and often caused them to miss clinic appointments as well as miss taking their medicines accordingly as they sought to balance access to care and other necessities of daily living such as securing food for their children as echoed in the following quote:

It is the issue of money. For me I fail to get money to transport myself to the facility to collect my drugs and yet these people (HWs) insist that I should continue getting drugs from the facility because I am not taking drugs well. And yet what makes me not pick drugs on my appointment dates is lack of transport, (34 years FSW, 7 years on ART).

#### Long waiting time

Although participants preferred to use FBIM DSD model, an issue that was frequently brought up in our study is the long waiting times at the health facility. This had negative effects on their job routines and their daily earnings:

Every time I come here (facility); we take so long yet I leave home when I have not prepared food for my children. I sometimes leave my ART card with the health workers and go back home to serve my children food due to the long waiting hours. Sometimes I fail to go back to collect drugs, I opt to do something productive which can earn me money than waiting for ART. In any case these days we get customers during the day because of Covid 19 lockdown, so I cannot just sit here the whole day while missing customers. (35 years FSW, 3 years on ART).

#### Community based models

##### Perceived lack of confidentiality

Many participants in this study expressed negative feelings about services provided through community-based models for fear of lack of or perceived lack of confidentiality among the group members in CCLADS. The gravity of the concern was deep such that participants confessed to avoiding accessing HIV care or shift to other health institutions if HWs insisted on them joining such models. Their concerns were specific to group members who may not keep secrets and disclose the HIV status of their group members including the HIV status of their children:

That cannot be possible, I can’t accept that. I can’t accept to be in that model (CCLAD)!.

Now look, me I may be taking Atiri-tiri (Atripra) when the other is taking Atanather, when gossiping, our very group member will say that those taking different line of drugs have stronger virus than the rest (39 years FSW, 15 years on ART).

They (CCLAD members) don’t even gossip about our HIV status only, they even talk about your other private things. Like I am married but also do sex work, imagine if someone told my husband and children!!! (30 years FSW, 4 years on ART).

##### Lack of privacy

The findings in this study bring the issue of privacy in community-based models to the spotlight. The lack of adequate space, clients lining up in open spaces as they wait to see HWs and selective provision of services that only target KP in the community-based models caused concerns of potential accidental disclosures. The participants’ biggest concern was that they would risk losing their partners and the imagination of having their children know that they are HIV positive and practice sex work at the same time. Such situations, they mentioned, psychologically affected them and their children risked being stigmatised.

That place (DIC) is known to provide HIV services to the KP community only, so people you know may see you going there, and they start spreading rumours in the community that you are HIV positive. For a person like me who sell snacks, if someone found me at the ART clinic, she may refuse to buy snacks from me because I am HIV positive, or she may tell a colleague not to buy anything from me. (35 years FSW, 2 years on ART).

I feel so bad being called weird names since I am a widow and stay with my children. I feel so bad if the community calls me an HIV positive prostitute because it can affect my children. I have shifted my home to many places because of such abuses. Once I notice that people are aware of the job I do, I just shift to another place, then how do you expect me to get my ART from the community!! (35 years FSW, 3 years on ART).

Some participants felt that the places in the community where services are provided did not have adequate space for privacy. They echoed their concerns in relation to inadequate private space:

If we could come and meet the doctor in the room, it would be much better compared to this one where we sit outside here as everybody who passes sees us. The place (FSW targeted outreach) is so open and exposed that even community members who are just passing see us and everybody here knows that these doctors come here to give drugs to sex workers. Imagine you already have discomfort with community members knowing that you are doing sex work and again they get to know that we are also HIV positive. We are so exposed. (26 years FSW, 2 years on ART).

##### Fear of disclosure

Participants expressed fear of disclosing their HIV status and type of work they do to their significant others by accessing services through community-based models. The following narratives reveal that they were afraid to lose both their stable partners and clients coupled with the possibility of negatively impacting their children through community stigma:

We are so much at risk of accessing ART from the community because most of us (FSWs) stay with our children in our communities and we may fear accessing those drugs in the presence of children. (45 years FSW, 7 years on ART).

Something that we do today will affect us in future in that even if you reach a time and quit the sex work job, people will not believe you. So, they may even discriminate against your children and even insult them that they are children of a sex worker. We suffer much criticism from the community members, and this becomes challenging to seek care from the service centre in my community (28 years FSW, 3 years on ART).

##### Lack of trust and conflicts in the community groups

Participants were uncomfortable trusting their colleagues with the drugs and even went ahead to make suggestions of how such models can be effectively managed. This discomfort was especially pinpointed in CCLADS, one of the community-based models, as indicated in the following quotes:

Joining client groups (CCLAD) wouldn’t be bad but they backbite us from there. I really wouldn’t want to engage in unnecessary conflicts, yet I already have bigger problems, that’s why I even stopped attending these groups. Those situations can stress you (30 years FSW, 4 years on ART).

Sometimes you may fail to trust a peer leader or a member in your ART group (CCLAD) to carry for you the ART drugs because she may put something evil or poison in the drugs. You see drugs are like food, how do you trust your exposed food to anybody. Moreover, the work we do we are ever competing for customers and some colleagues are jealousy when you get many rich customers. In our places of work there is a lot of Juju (witchcraft) because of competition for customers. May be HWs should put branded seals for each person’s drugs, as for me I can never send anyone to pick my drugs (23 years FSW, 3 years on ART).

## Discussion

In this study we sought to find out FSWs’ perspectives on differentiated service delivery models. DSD models for female sex workers are scaled up with the goal of expanding access to HIV services and treatment continuity. However, there is limited research on FSWs’ preferences, facilitators, and barriers to the utilization of DSD models. Key findings from this study indicate that the FBIM model was most preferred due to a wide array of comprehensive health services provided, confidentiality, privacy, and the availability of professional health workers at facilities. On the other hand, there was substantial interest in receiving care from community-based DSD models due to easy services access, reduced transport costs, the flexibility in working hours at DICs and the benefit of interacting with more friendly health workers in community-based DSD models especially DICs. However, the community-based models were least preferred due to stigmatization and discrimination in the community, lack of privacy and confidentiality, and a limited package of health services offered. Targeted strategies to reduce stigma and discrimination and the provision of comprehensive high-quality services have potential to optimise FSWs’ access to HIV services through community-based DSD models.

The participants provided mixed reactions to the different models and FSWs were specifically apprehensive about bringing services to the communities where they work and live due to fear of stigmatization and discrimination by community members. Most of the participants preferred to access HIV care services from the facilities using an FBIM model mainly because of the benefit of access to free comprehensive health services and being able to be attended to by professional health workers as well as the privacy and confidentiality at health facilities. The participants however were quick to suggest that clinics would have to be reorganised to reduce waiting time that is currently long, and also reduce the frequency of drug pick-ups and clinic reviews. In South Africa and Uganda [3, 24], it was previously documented that PLWHIV expressed a preferential access to care from established clinic settings rather than community settings if they were given more months before drug refill is due. As part of improving client-centred services, WHO [2] and PEPFAR [25] promote dispensing of ART drugs to last for three and more months. Furthermore, they encourage spacing of appointments, thus what participants propose in our study should be feasible to put into consideration after understanding each client’s needs, preferences, and behaviours.

Services such as constant condom availability, STI treatment, provision of contraceptives, screening for hepatitis B, viral load sample collection were reportedly not routinely provided through the community-based models. Other studies have reported limited HIV care package provided at community level and the impact this may have on health outcomes [15, 24, 26]. Expanded service packages at community level will increase confidence among clients and potential increase in utilisation of services throughout facility DSD models and thus improved retention in care [3]. Even though, studies have reported that similar health outcomes have been observed among clients in community-based and facility-based ART delivery models [27, 28] programs should not just assess clinical outcomes but also consider processes of service delivery since these contribute to the good clinical outcomes as important markers to continuity in HIV care.

This study has shown that FSWs were specifically concerned about the lack of privacy in community-based models. Privacy concerns have been reported in other studies in community-based DSD models, for example a recent assessment on community-based ART service model of FSWs in Malawi [29] reported that most FSWs faced high external stigma after the community members learnt of their HIV positive status. In contrast, the same report and other studies in Zimbabwe and Benin, have indicated that DICs are more appealing to FSWs because of the privacy they offer over health facilities, as the DICs are often not crowded and have more friendly HWs [17, 29, 30]. This means that DICs could also be one of the preferred DSD models, if there was a deliberate effort to carefully set up DICs in locations that maximise privacy since in this study privacy concerns were mainly on setting, space, and location while FSWs may not have an issue of privacy while already inside the DIC.

Another key finding was the concern in the lack of confidentiality especially in DSD models such as CCLADs and FBGs which involve interaction at group level. Relatedly, participants reported lack of trust and conflicts in the group DSD models and were uncomfortable trusting their colleagues with their drugs. The conflicts were attributed to failure to maintain HIV status confidentiality, rumour mongering about private issues, use of stigmatising statements on colleagues and poor management of CCLADs and FBGs. The lack of confidentiality and trust issues in group DSD models has been reported in other studies in Malawi and Tanzania as a major contributor to failure of such types of DSD models [31, 32]. This issue of confidentiality touches on a broader base of professional practice and the ethics in the health care system. While there is a structured plan in the health care system to address confidentiality in health care access, the plan largely focuses on professional health workers. Professional HWs are often trained on confidentiality of patient information and policies and guidelines for use by HWs [2, 33–35]. The changing dynamic of engaging FSW peers and patient support groups presents a new challenge in observing confidentiality, pointing to the importance of formally enhancing understanding of service delivery ethics for anyone engaged in informal service delivery. While there have been efforts to train the peers that are formally engaged by facilities, community health workers and village health teams [35, 36], the trainings are not structured to follow a routine training plan. Currently there is no systematic way of getting patient groups that are involved in picking drugs in DSD models such as CCLADs, yet they need to know the sensitivity of confidentiality when dealing with patients’ information. To address barriers related to confidentiality, strategies such as training of peers and patient groups, use of confidentiality agreements for stakeholders and developing simplified confidentiality reference materials, disseminating them, and displaying posters focusing on proper discharge of confidentiality in service delivery areas is an important factor for the successful implementation of group DSD models.

Furthermore, we also demonstrated that while most of the participants preferred to access care at health facilities, overall, there was substantial interest in receiving care from community based DSD models due to easy services access, reduced transport costs, the flexibility in working hours at DICs and the benefit of interacting with more friendly health workers in community-based models especially DICs. This points to the confidence participants would likely have in community-based models if the concerns regarding privacy, confidentiality, community stigma and discrimination are addressed. On the other hand, it may be a point on how strongly clients desire to be fully assessed by health workers and receive a comprehensive package of services at facility level such that they must alternately seek care services from both models of care. Previous studies done in Tanzania, Zimbabwe, Uganda, and Kenya [24, 29, 37, 38], also reported that implementation of comprehensive and enhanced package of care at community level that is managed by trained and friendly health workers leads to an increase in access to services and improved health outcomes. However, stigma and discrimination are complex to manage, and it was consistently cited as a barrier to community DSD utilisation. Similarly, other studies have also identified stigma and discrimination as a serious impediment in access to HIV services in community HIV based service delivery [39, 40]. This implies that there is need to develop interventions to address internalized and perceived stigma and discrimination as well as engage in further research in the context of community based DSD models. Such interventions could include counselling and supporting FSWs to weigh the benefits of accepting their behaviour and receive appropriate and timely health services. This might help them overcome stigma and safeguard against stress and fear when accessing health services from a community based DSD model. Furthermore, results from this study indicate a need to address privacy issues related to the physical locations where community-based DSD models are provided. Therefore, identifying the setting, space and locations with maximum privacy could help FSWs overcome internalized stigma and feel comfortable to access services freely through community-based DSD models.

Our study has some limitations. FSWs in this study were drawn from only two public health facilities and two DICs, therefore their perspectives may not accurately represent other FSWs accessing care from other treatment centres. In addition, we did not interview adolescent FSWs living with HIV and therefore our findings may not reflect the perspectives of this more vulnerable FSW sub-population. Further, other important aspects in differentiated care were not explored such as sustainability that could have a huge impact on DSD models when PEPFAR funding ceases. Lastly, the participants of this study were FSWs, a marginalised population that may not freely express their perceptions and feelings in the society. While we tried to ensure quality data is collected by interviewing only those that had expressed willingness to their gatekeepers, and created a friendly environment with good rapport, we cannot rule out the fact that FSWs may have withheld some information.

## Conclusion

This study has provided critical data based on FSWs’ perspectives on factors that may contribute to the successful implementation of the DSD models. Although existing evidence suggests that community-based DSD models are easily accessible, reduce opportunity costs for FSWs and have optimal peer support, we found that FSWs mostly preferred facility based DSD models due to the benefit of access to free comprehensive health services and being able to be attended to by professional health workers as well as the privacy and confidentiality at health facilities. Fear of stigma and discrimination in the community, lack of privacy, lack of confidentiality and limited packages of HIV services were major barriers to accessing community-based DSD models. In view of the study findings elucidated in this paper, the following recommendations are made; that there is need to expand and improve the capacity of community-based DSD models to provide a one stop shop comprehensive package of HIV services including a wider range of FSWs targeted health services. This study indicates a need to ensure constant availability and provision of services such as sufficient condom supply, STI testing and treatment, provision of contraceptives and screening for hepatitis B. In addition, collection of HIV viral load samples in the community could increase access of services through the community-based models*.* Further, at facility level, deliberate efforts such as extending clinic operating hours, flexibility in service provision arrangements such as waiving off mandatory treatment buddy at enrolment and provision of non-judgmental and friendly services will go a long way to address access to HIV services and retention in care for FSWs. There is also need to develop holistic interventions to curb stigma and discrimination among FSWs subpopulation and the community and those interventions should be inclusive of structural as well as institutional aspects of health. Lastly, there is need for further studies to focus on community based DSD models for FSWs to provide in-depth and relevant perspectives on how the service delivery models would be improved to cater for FSWs living with HIV in various settings. The findings from such studies would help to inform HIV policy makers on the best approaches to the implementation of DSD models in various contexts.

## Data Availability

Due to conditions of ethical approvals of research among key populations, we are unable to provide access to the full dataset on a public repository. However, we are willing to de-identify transcripts and make them available upon reasonable request. Interested persons should contact the corresponding author.

## Abbreviations

ART: Antiretroviral therapy
CCLAD: Community client led art delivery
CDDP: Community drug distribution point
DSD: Differentiated service delivery
DIC: Drop-in center
FBG: Facility based groups
FTDR: Fast track drug refills
IDI: In-depth interviews
KP: Key population
PLWHIV: People living with HIV
UVRI: Uganda Virus Research Institute
WHO: World Health Organization
